# Extensive epithelioid hemangioendothelioma of the maxillary sinus: A case report

**DOI:** 10.1016/j.ijscr.2019.04.013

**Published:** 2019-04-16

**Authors:** A. Chaouki, A. Mkhatri, A. Ballage, N. Zouhair, M. Mahtar

**Affiliations:** ENT Department, 20 August Hospital, CHU Ibn Rochd, Casablanca, Morocco

**Keywords:** Epitheloid haemangioendothelioma, Maxillary sinus, Radiation therapy, Chemotherapy

## Abstract

**Background:**

Epitheliod hemandioendothelioma (EHE) is a rare vascular tumor which was first reported by Weiss and Enzinger in 1982. It can be seen in many locations whose paranasal sinus is extremly rare. Its main treatment is surgery. To our best knowledge, no report of EHE treated by radiation therapy and chemotherapy has been described.

**Case summary:**

A 18 years old man presented to our hospital with a 3 months history of right intermittent epistaxis, permnanent nasal obstruction and right hearing loss. The physical exam found a right exophtalmia, swelling deformatted right hemifacia and a bulky whitish tumor filling the right nasal cavity. The magnetic resonance imaging (MRI) showed an extensive hyperascularized nasopharyngeal process filling the right nasal cavity whose histopathological exam revealed an epitheloid hemangioendothelioma. Due to intracranial extension, surgery was contraindicated. The patient received 65 Gy of radiation therapy and chemotheray (cisplatin). After 18 months of follow up, the exopthalmous regressed and the MRI showed a 50% regression of the tumour size.

**Conclusion:**

Extensive EHE can be treated by radiation therapy and chemotherapy when surgery can’t be perfomed.

## Highlights

• Epithelioid haemangioendothelioma is a rare vascular tumor which can originate from the maxillary sinus.
• Surgical excision is the treatment of choice for local tumors.
• Exclusive radiation therapy with chemotherapy can be an option for extensive forms.

## 1 Introduction

Epithelioid hemangioendothelioma (EHE) is an uncommon vascular tumor of soft tissue, which was first reported by Weiss and Enzinger in 1982. This unusual vascular neoplasm is characterized by proliferation of a distinct type of endothelial cells which show epithelioid morphology.

This rare tumor with an estimated incidence of approximately one person in every 1.000.000 have histological and clinical features intermediate between those of benign hemangioma and conventional angiosarcoma.

Due to the rarity and histological similarity to other tumors, diagnosis can be challenging [1].

There are very few cases of EHE of nasal cavity described in the English literature [2]. We describe here an advanced case of EHE of maxillary sinus in a young man who was treated by radiation therapy and chemotherapy. This work is reported in line with SCARE criteria [3].

## 2 Case report

**Fig. 1:**
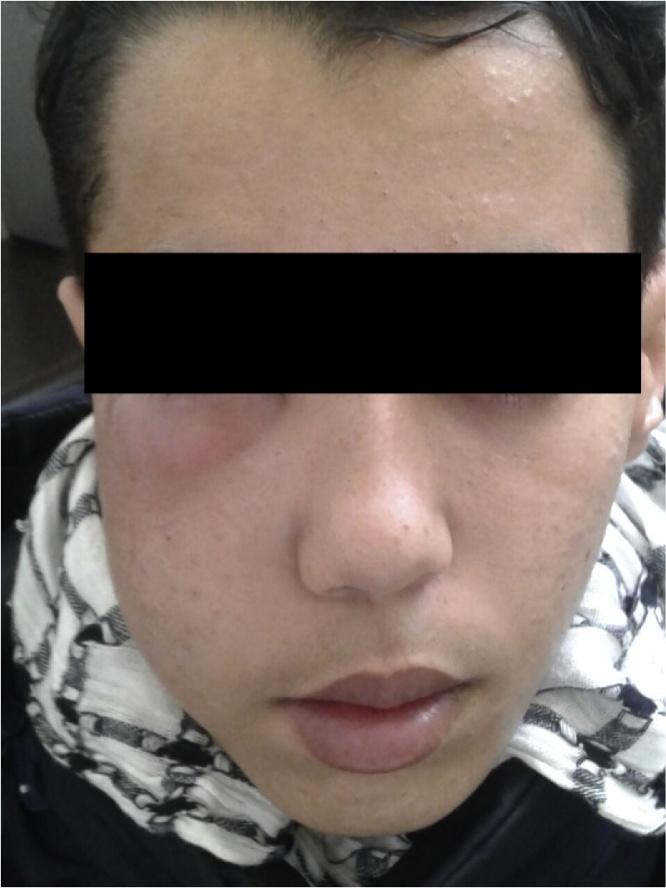
Patient admitted with deformated right hemiface with exopthalmos.

A 18-year-old man presented with a 3 months history of right intermittent epistaxis, permanent nasal obstruction, anosmia and right hearing loss. No other nasal or ocular symptoms were noticed. The physical exam found a right exophtalmia, a swelling deformatted right hemiface (Fig. 1) and a bulky whitish tumor filling the right nasal cavity at the nasal endsocopy. Cranial nerves, neck and oral cavity exams were normal.

**Fig. 2:**
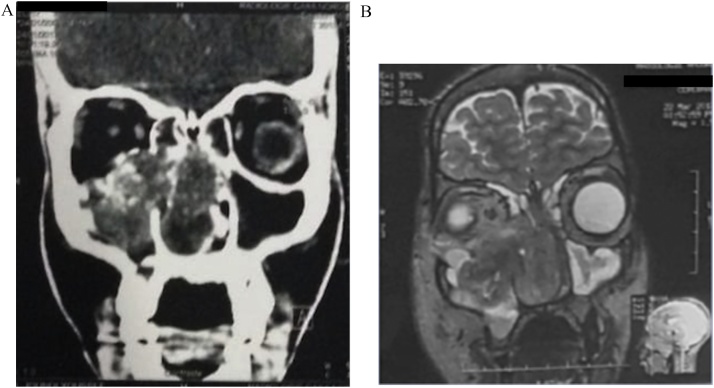
Imaging findings in CE-CT and MRI. (A) CT showed heteregenous tumor mesuring 8.7 × 6.5 cm, heterogenously enhancing with important lysing of the inner wall of the right orbit. (B) MRI showed confirmed high vascularized and extensive mass filling the right nasal cavity.

A computed tomography showed heterogeneous tissue tumor, measuring 8.7 × 6.5 cm heterogenously enhancing. Important lysis of the inner wall of the right orbit with important extension (intra-orbital, intra sellar, nasopharynx and right pterygoide fossa) (Fig. 2).

Magnetic resonance imaging (MRI) confirmed the orbital, intra sellar and the right pterygoide fossa extension with a hyper vascularized nasopharyngeal process filling the right nasal cavity (Fig. 2).

**Fig. 3:**
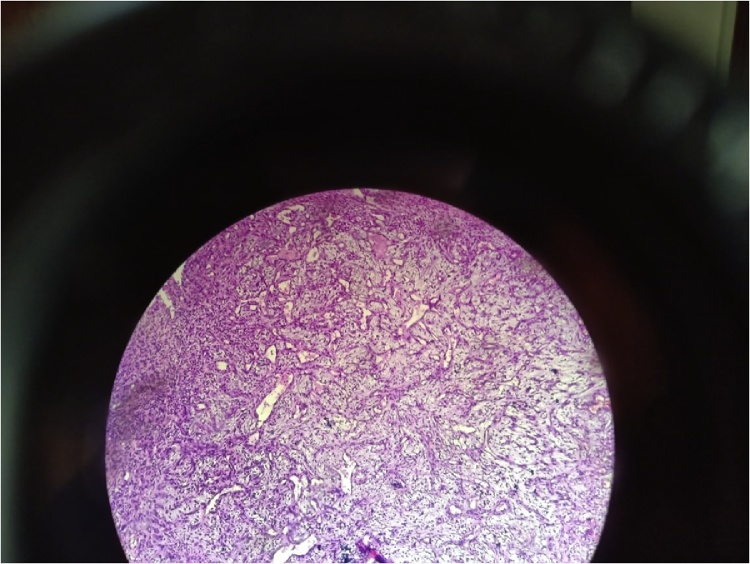
Ulcerated tumor proliferation richly vascularized with thickened-wall vessels and turgid endothelium. This proliferation is made of globular cells with abundant eosinophilic cytoplasm. This aspect is compatible with epitheloid hemangioendothelioma.

Microscopic examination of the biopsy showed ulcerated tumor proliferation richly vascularized with thickened-wall vessels and turgid endothelium. This proliferation is made of globular cells with abundant eosinophilic cytoplasm. The nuclei are moderately hyperchromatic sites of moderate cytonuclear atypia. This proliferation is dissociated by lymphoplasmocytes and histiocytes. Immunohistochemical study shows cytokeratin negativity and positivity of CD31. The histological aspect is compatible with a epitheloid hemangioendothelioma (Fig. 3).

Due to aggressivity, advanced tumor stage and intracranial extension, the surgery was contraindicated.

The patient received 55 Gy of intensity modulated radiotherapy (IMRT) with weekly chemotherapy made of cisplastine (40 mg/m^2^) for 6 weeks.

**Fig. 4:**
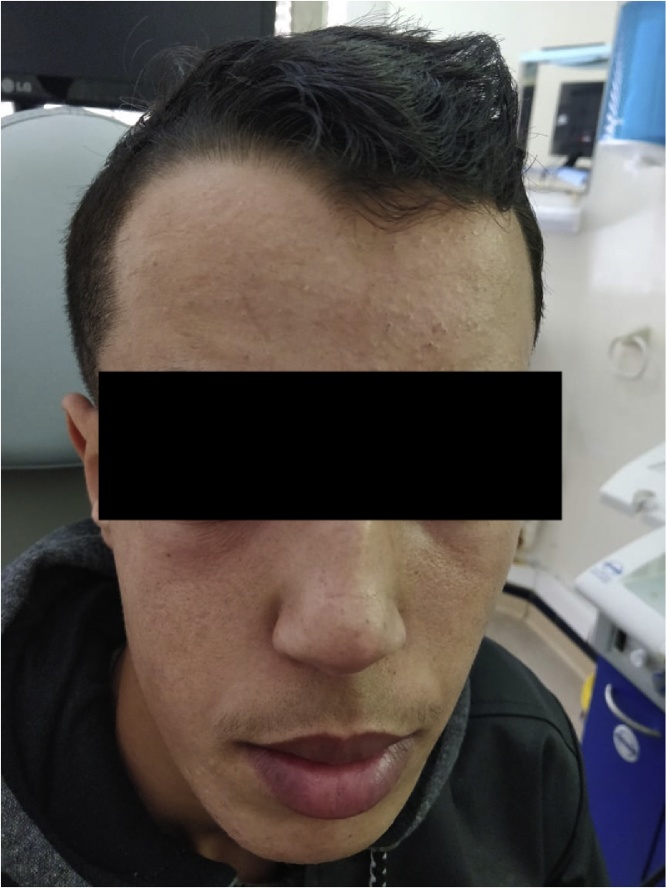
Important regression of the deformation of the right hemiface and the exopthalmous 18 months after admission.

**Fig. 5:**
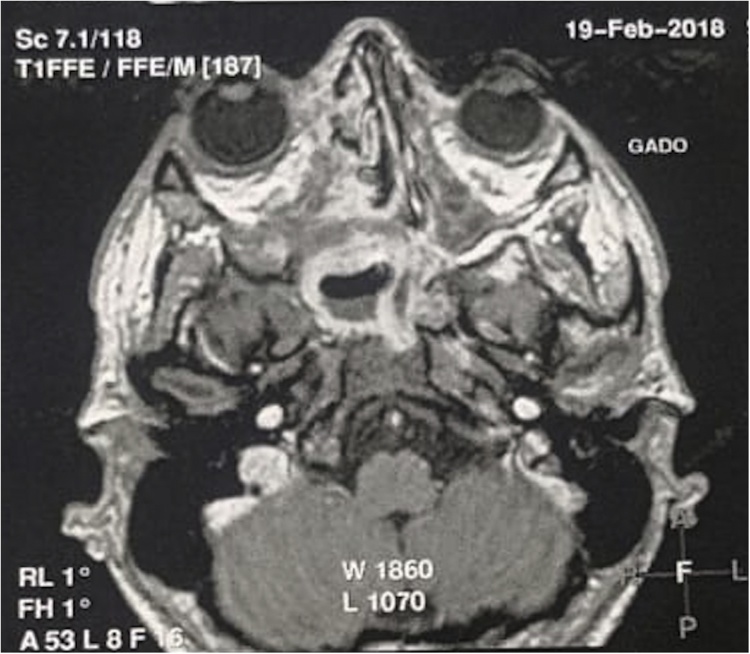
Axial image of MRI showing regression of 50% of the tumor after radiation therapy and chemotherapy.

At 18 months follow-up, the exopthalos regressed (Fig. 4) and the MRI showed 50% regression of the tumor process (Fig. 5).

Otherwise the patient presented a mucositis of the right cheek, cured by medical treatment.

## 3 Discussion

EHE was first described by Weiss and Enzinger in 1982 as a tumor that affects soft tissue [1]. It is an angiocentric vascular tumor with endothelial component. There is no sex predilection and age of onset is variable [4].

It can occurs in several locations: soft tissue, liver, lung, bone, skin, oral cavity, gastrointestinal tract, peritoneum, lymph nodes, meninges, pleura, heart, mediastinum, spleen, thyroid or parotid gland [5].

The nasal cavity is also an extremely rare location for this tumor, with only 7 cases reported in the literature since 2016 [6]. Even rarer, however, is an EHE primarily originating within a paranasal sinus. To our best knowledge, only two cases of EHE originating in the maxillary sinus has been previously described in the literature [7,8].

Both by clinical as by histological characteristics, EHE is considered as a tumor of intermediate malignancy because of both local recurrences and metastatic potential [6].

The tumor cells often develop as a painful mass in either superficial or deep soft tissue. On gross inspection, the tumor usually has a variegated, white red color that offers a hint of its vascular nature [3].

Histologically, the tumor is composed of short strands or solid nests of rounded to slightly spindled endothelial cells. Some canalized vascular channels and clusters of erythrocytes are seen.

Immunohistochemically, these tumors are positive for cytokeratin (especially CK18), and vascular markers including CD31. These tumors have an intermediate and variable malignancy pattern in terms of both local recurrences and metastatic potential [8].

Therefore, wide local excision is considered the treatment of choice, and is probably curative in most cases. In a few cases, adjuvant treatment with radiotherapy or chemotherapy has been given [3].

In our case, it was local aggressive EHE of the maxillary sinus in a young man with intracranial extension, treated with IMRT with weekly chemotherapy. To our best knowledge, this is the first EHE treated only with radiation therapy and chemotherapy.

Given the limited clinical data, evidence based treatment protocols for treatment for sinonasal EHE are not possible. Therefore it requires a close clinical follow-up because of its potential to recur and metastasize [8].

After 18 months follow-up, the evolution was good with regression of the exophthalmos and 50% of the tumor size.

## 4 Conclusion

EHE is an uncommon tumor of soft tissue whose nasal cavity is extremely rare but should be considered in the differential diagnosis of a nasal cavity mass. Surgical excision is the treatment of choice when limited to the nasal cavity. Radiation therapy with chemotherapy can be an option for EHE particularely when it is extensive.

## Conflicts of interest

The authors declare that they have no competing interests.

## Sources of funding

This research did not receive any specific grant(s) from funding agencies in the public, commercial, or not-for-profit sectors.

## Ethical approval

I certify that this kind of manuscript does not require ethical approval by the Ethical Committee of our institution.

## Consent

Written informed consent was obtained from the patient for publication of this case report and accompanying images. A copy of the written consent is available for review by the Editor-in-Chief of this journal on request.

## Author’s contribution

All authors were involved in the care of the patient.

Anass Chaouki was involved in study conception, design and acquisition of data.

Amine Mkhatri and Amine Ballage were involved in drafting of manuscript.

Najib Zouhair and Mohamed Mahtar was involved in critical revision.

## Registration of research studies

We do not have the funds to register this study. We remind that Morocco is eligible for group A countries for the free access about APCs.

## Guarantor

Dr Anass Chaouki.

## Provenance and peer review

Not commissioned, externally peer-reviewed.
